# Invasion history and demographic pattern of *Cryphonectria hypovirus 1* across European populations of the chestnut blight fungus

**DOI:** 10.1002/ece3.429

**Published:** 2012-11-22

**Authors:** Sarah F Bryner, Daniel Rigling, Patrick C Brunner

**Affiliations:** 1 WSL Swiss Federal Research InstituteCH-8903, Birmensdorf, Switzerland; 2 Institute of Integrative Biology, ETH ZurichCH-8092, Zurich, Switzerland

**Keywords:** Biological control, coalescent analysis, *Cryphonectria parasitica*, host–parasite coevolution, phylogenetic reconstruction, time to the most recent common ancestor

## Abstract

We reconstructed the invasion history of the fungal virus *Cryphonectria hypovirus 1* (CHV-1) in Europe, which infects the chestnut blight fungus *Cryphonectria parasitica*. The pattern of virus evolution was inferred based on nucleotide sequence variation from isolates sampled across a wide area in Europe at different points in time. Phylogeny and time estimates suggested that CHV-1 was introduced together with its fungal host to Europe and that it rapidly colonized the central range along the south facing slopes of the Alps and the north-east facing slopes of the Dinaric Alps. These central populations were the source for two waves of simultaneous invasions toward the southern Balkans and Turkey, as indicated by migration rates. Our results showed that the evolutionary scenarios for CHV-1 and *C. parasitica* were spatially congruent. As infection with CHV-1 reduces the pathogenicity of *C. parasitica* toward the chestnut tree, CHV-1 invasions of the newly established *C. parasitica* populations probably prevented the development of devastating chestnut blight epidemics in Europe. We propose that in this, and supposedly in other pathosystems, geographic, vegetation-related, demographic, economic, and political factors may help explain the correlated invasion pattern of a parasite and its host.

## Introduction

In modern time, global travel and trade have repeatedly facilitated the introduction of foreign species and their establishment in new environments (Perrings et al. 2002). In many cases, such introductions have promoted the emergence of new diseases (Anagnostakis 1987; Parker and Gilbert 2004). The population dynamics of parasites, in particular, of obligate parasites such as viruses, depends largely on the dispersal of the host (Biek and Real 2010). The pattern of virus evolution can therefore be spatially and/or temporally congruent with the evolution of the host (Holmes 2004). The majority of DNA viruses are persistent, transmitted vertically (to offspring) or sexually, and their virulence is often low (Villarreal et al. 2000; Holmes 2008). They have an evolutionary rate similar to that of their host and their evolution is mostly congruent with that of their host on both the spatial and the temporal scale. The vast majority of RNA viruses, in contrast, cause acute (i.e. short-term) infections, are transmitted horizontally from host to host or by vectors, and are usually highly virulent. RNA viruses have fast nucleotide substitution rates, largely exceeding that of their hosts, they evolve rapidly and may even jump species barriers (Villarreal et al. 2000; Holmes 2008). The evolution of acute RNA viruses is therefore rarely congruent with the evolution of their hosts on the temporal scale (Villarreal et al. 2000). Nevertheless, RNA viruses that depend on horizontal transmission between hosts have often the same geographic distribution as their host, and thus show an evolutionary pattern congruent with that of their host on a spatial scale (Real et al. 2005; Biek et al. 2007; Nadin-Davis et al. 2010; Torres-Pérez et al. 2011).

**Figure 1:**
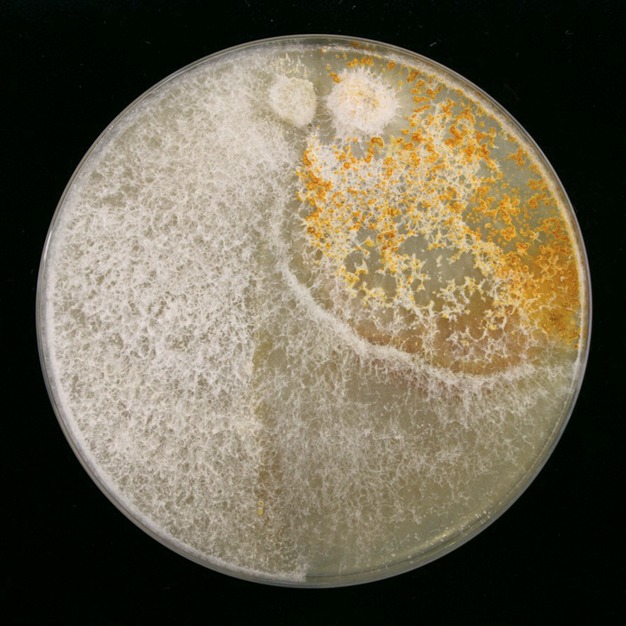
Two fungal cultures of *Cryphonectria parasitica* were grown on potato dextrose agar. Initially, the culture on the left harbored *Cryphonectria hypovirus 1* (white color) and the culture on the right was virus-free (orange color). While grown on the same Petri dish, the virus was transmitted from the culture on the left to the culture on the right, which then also took on a white color at the bottom (newly grown mycelium).

*Cryphonectria hypovirus 1* (CHV-1) is an RNA virus that persistently infects its host, the fungus *Cryphonectria parasitica*. It is therefore an exception among RNA viruses causing short-term infections in their hosts, and also among fungal viruses. Unlike the vast majority of fungal viruses, CHV-1 is virulent and causes marked symptoms in the fungus (Nuss 2005). CHV-1 does not kill its host, but it inhibits sexual reproduction, attenuates growth, and strongly reduces asexual sporulation of the fungus (Milgroom and Cortesi 2004). Spread of CHV-1 depends on a combination of vertical and horizontal transmission. CHV-1 is transferred into the asexual spores of the fungus (vertical transmission), dispersed in these spores and then transmitted from the outgrowing spores to other fungal individuals (horizontal transmission) by hyphal fusion (Fig. 1; Hoegger et al. 2003; Milgroom and Cortesi 2004). However, asexual spores are only dispersed over short distances, unless they are carried by vectors such as insects, birds, mammals or by humans on chestnut plants or wood (Heiniger and Rigling 1994). Spread and dissemination of CHV-1 is therefore expected to depend largely on host movement and contacts between hosts.

The host of CHV-1, the fungus *Cryphonectria parasitica*, is native to East Asia and was introduced to North America and Europe early in the 20th century (Anagnostakis 1987). *C. parasitica* is a serious tree pathogen and causes lethal bark cankers on susceptible chestnut (*Castanea* spp.). After its introduction to Italy, either directly from Asia or via a bridgehead in North America (Dutech et al. 2012), it spread rapidly throughout the chestnut growing regions and evoked a dramatic chestnut blight epidemic (Anagnostakis 1982). In North America, it destroyed the native chestnut forests, whereas in Europe, chestnut blight incidence to date is high, but maintained at low severity. The slowdown of the epidemic in Europe may have – at least in part – resulted from infection of *C. parasitica* with CHV-1 and the emergence of this debilitating viral disease in the fungal population (Heiniger and Rigling 1994; Milgroom and Cortesi 2004). CHV-1 occurred naturally and spread spontaneously in European *C. parasitica* populations, and has thus been biologically controlling the disease. Infection with CHV-1 reduces the pathogenicity of *C. parasitica* to the chestnut tree, a phenomenon called hypovirulence (Van Alfen et al. 1975; Nuss 2005). Much research on chestnut blight has been directed toward the characterization of fungal populations based on the diversity of vegetative compatibility (vc) types (Cortesi et al. 1998; Milgroom and Cortesi 1999; Sotirovski et al. 2004; Braganca et al. 2007; Krstin et al. 2008; Milgroom et al. 2008; Montenegro et al. 2008; Robin et al. 2009; Dutech et al. 2010). *C. parasitica* vc types are determined by at least six bi-allelic genetic loci and can therefore be used as a measure for genetic diversity (Cortesi and Milgroom 1998). Milgroom et al. (2008) found that *C. parasitica* populations in the central European range were genetically diverse, while the more recently founded populations in the southern Balkans and in Turkey were mainly clonal. Furthermore, the data indicated that the populations in the southern Balkans and in Turkey had been the result of two independent invasion events.

Since CHV-1 has been found in China and Japan, it has been assumed that CHV-1 was introduced together with *C. parasitica* from Asia (Liu et al. 2007). Records of first detection of hypovirulent *C. parasitica* in different regions indicated that CHV-1 spread throughout Europe following the spread of the fungus with a lag in time (Heiniger and Rigling 1994; Robin and Heiniger 2001). The dependence of CHV-1 on its host for spread and transmission as described above may further support the suggestion of a tight relationship between spread of the fungus and spread of the virus. However, while many studies were directed at studying the fungal host population (Cortesi et al. 1998; Milgroom and Cortesi 1999; Sotirovski et al. 2004; Breuillin et al. 2006; Braganca et al. 2007; Krstin et al. 2008; Milgroom et al. 2008; Montenegro et al. 2008; Robin et al. 2009; Prospero and Rigling 2012), the genetic structure of CHV-1 populations in Europe has remained largely unexplored. Therefore, information about the dispersal of CHV-1 is based on first records of CHV-1 in the different European regions (Heiniger and Rigling 1994; Robin and Heiniger 2001) and little is known about the true population structure of CHV-1. Four genetically distinguished subtypes of CHV-1 have been described in Europe, named subtype I, D, F1, and F2 (Allemann et al. 1999; Gobbin et al. 2003). The degree of genetic differentiation together with the estimated rate of nucleotide substitution suggested that these subtypes diverged several hundred years ago (i.e. before the introduction of the fungus) and that they were introduced at independent events to Europe (Gobbin et al. 2003). Among the four subtypes, subtype I is the most widespread and is prevalent all across Switzerland, Italy, south-eastern Europe and Turkey, thus, providing the opportunity to study the invasion of this subtype across the expanding range of its fungal host.

To our knowledge, there is only one molecular study of CHV-1 in Europe, which investigated virus transmission within and between two *C. parasitica* populations in Italy (Carbone et al. 2004). Here, we generated a large data set of CHV-1 sequences from Europe by sampling eight populations from different geographic regions. By complementing this recent data set with CHV-1 sequences obtained from earlier samplings, we were able to reconstruct the unexplored phylogenetic history of CHV-1 in Europe. We tested the hypothesis of evolutionary congruence between the virus and its fungal host, estimated the time to the most recent common ancestor (TMRCA) using coalescent analysis, and discussed the likely pattern of invasion.

## Materials and Methods

### Samples of CHV-1

In 2008/2010, we sampled a total of eight virus-infected *C. parasitica* populations that were naturally infected with CHV-1 (Gobbin et al. 2003). All fungal populations were sampled within an area of <1 ha. Four of these populations were obtained from the central range (Switzerland and Bosnia-Herzegovina) where vc type diversity was known to be high and another four populations were obtained from the southern range (Macedonia, Greece and Turkey) where *C. parasitica* populations were dominated by a single vc type (Robin and Heiniger 2001; Milgroom et al. 2008). All sampling sites were coppice forests with 10–20 years old chestnut sprouts and a high incidence of chestnut blight. Bark samples were taken with a cork borer (5 mm diameter) from chestnut blight cankers at intervals of at least 5 m between trees. Only one canker per tree was sampled and the cork borer was sterilized with 70% ethanol and flaming between cankers. We obtained pure cultures of *C. parasitica* by isolation from the bark samples on water agar followed by culturing on potato dextrose agar (Difco Laboratories, Detroit, MI, USA; Robin et al. 2010). The vc type of each *C. parasitica* isolate was determined by pairing with vc type tester strains (Cortesi and Milgroom 1998). The viral double-stranded (ds) RNA was extracted from lyophilized mycelium by cellulose CF-11 chromatography as described by Allemann et al. (1999).

In addition to the eight CHV-1 subtype I populations described above, we extended our data set by including sequence data from two populations sampled in 1998 from the central host range (one from Switzerland and one from Bosnia-Herzegovina) and data for two populations sampled in 2000 from the southern range (Macedonia). We also added a few individual CHV-1 sequences from Europe dating back to the 1970s, including the reference sequences for CHV-1 subtype I (CHV-1/Euro7) and CHV-1 subtype F1 (CHV-1/EP713) and additional samples from each of the four CHV-1 subtypes published in an earlier study (Gobbin et al. 2003). These older samples were kept either freeze-dried or in glycerol stocks at −80°C to avoid subculturing. Finally, we included Genbank sequences from China and Japan, the putative origin of CHV-1. An overview over all populations and individual samples used in this study is given in Table 1.

**Table 1:** *Cryphonectria hypovirus 1* samples used in this study

Country	Year of sampling	Name, location	Population	Number of samples	Reference [GPS coordinates]
Switzerland	2010	Pu, Pura	Yes	42	This study [45.98 N, 8.86 E]
Switzerland	2010	Go, Gnosca	Yes	50	This study [46.24 N, 9.01 E]
Macedonia	2010	Ra, Radolista	Yes	55	This study [41.16 N, 20.62 E]
Greece	2010	He, Anelio	Yes	41	This study [39.43 N, 23.14 E]
Turkey	2010	Bu, Kurşunlu	Yes	33	This study [40.36 N, 29.02 E]
Turkey	2010	Ya, Kurtköy	Yes	17	This study [40.58 N, 29.22 E]
Bosnia-Herzegovina	2008	Ka, Kostajnica	Yes	38	This study [45.22 N, 16.55 E]
Bosnia-Herzegovina	2008	Iv, Ivanjska	Yes	30	This study [44.88 N, 17.04 E]
Switzerland	1998	CHF, Faido	Yes	17	(Gobbin et al. 2003)
Bosnia-Herzegovina	1998	BOVr, Vrnograc	Yes	18	D. Rigling (unpubl. data)
Macedonia	2000	MKVrat, Vratnika	Yes	10	D. Rigling and K. Sotirovski (unpubl. data)
Macedonia	1996	MKFra, Frangovo	Yes	2	D. Rigling and K. Sotirovski (unpubl. data)
Switzerland	1976	E-3	No	1	Gobbin et al. (2003)
	1976	E-5	No	1	Gobbin et al. (2003)
	1976	E-6	No	1	Gobbin et al. (2003)
	1980	E-27	No	1	Gobbin et al. (2003)
Italy	1978	CHV-1/Euro7	No	1	Chen and Nuss (1999)
France	1970	CHV-1/EP713 (F1)	No	1	Shapira et al. (1991)
	1970	E-55 (F1)	No	1	Gobbin et al. (2003)
	1974	E-56 (F1)	No	1	Gobbin et al. (2003)
	1975	E-57 (F2)	No	1	Gobbin et al. (2003)
	1997	E-62 (F1)	No	1	Gobbin et al. (2003)
Spain	1988	E-71 (D)	No	1	Gobbin et al. (2003)
Germany	1992	E-72 (D)	No	1	Gobbin et al. (2003)
China	1987–2002	CN	No	11	Genbank accession numbers HM246637 to HM246647
Japan	1992	JP	No	2	Genbank accession numbers HM246649 and HM246650

CHV-1; *Cryphonectria hypovirus 1*.

### Sequencing of CHV-1

Complementary DNA (cDNA) was synthesized from the dsRNA with random hexamer primers using the Maxima First Strand cDNA Synthesis kit from Fermentas (St. Leon-Rot, Germany). PCR amplification and sequencing were performed as described in Gobbin et al. (2003) with the modification that EP721-4 (5′- GGAAGTCGGACATGCCCTG-3′) was used as reverse primer. This allowed us to sequence a variable 693 bp region of open reading frame A corresponding to positions 1473–2165 in the nucleotide sequence of CHV-1/Euro7 (Chen and Nuss 1999).

### Haplotype determination and diversity measures

CHV-1 haplotypes were determined in DnaSP v5 (Librado and Rozas 2009) and haplotype richness (R_Haplo_) was calculated for each population. The Shannon–Wieners index was used as an estimate for the diversity of haplotypes (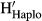). Diversity (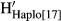) and richness (R_Haplo[17]_) of haplotypes expected for the smallest sample size being analyzed (*n* = 17) was determined by rarefaction analysis implemented by the vegan package in the software R 2.6.2 (R-Development-Core-Team 2008). DnaSP was further used to estimate nucleotide diversity (π) in each CHV-1 population. In addition, richness (R_Vc_) and diversity (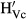) of vc types were calculated as a measure for diversity of the fungal host populations and rarefaction analysis was employed to determine richness (R_Vc[44]_) and diversity (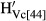) expected for the smallest sample size analyzed (*n* = 44). After rarefaction, independent samples *t*-test in SPSS 19.0 (SPSS, Somers) was employed to detect significant differences in richness and diversity among virus and among fungus populations.

### Bayesian coalescent analyses

We used the program BEAST v1.6.1 (Drummond and Rambaut 2007) to conduct coalescent-based genetic analyses. The program consists of a Bayesian Markov chain Monte Carlo inference package and a range of coalescent models. The input file was generated with help of the BEAUTi program implemented in the BEAST package and sequences were dated with the year of sampling (see Table 1). Our full set of samples obtained at different points in time allowed us to estimate the evolutionary rate and the time of divergence between evolutionary lineages, that is the TMRCA.

All analyses were performed using the HKY85 substitution model (Hasegawa et al. 1985) with gamma correction for site rate heterogeneity as determined by MODELTEST (Posada and Crandall 1998).

We ran preliminary BEAST analyses to select the most appropriate molecular clock (strict vs. relaxed) and coalescent population model (constant size, exponential growth, Bayesian sky line) based on Bayes factors (*K*) comparisons (Kass and Raftery 1995). BEAST was run for 10^8^ generations with three repeats to ensure convergence. The TRACER v1.5 program (Rambaut and Drummond 2007) was used to combine the BEAST output log-files for the final analysis and to calculate Bayes factors for model selection.

We inferred the population dynamic of the virus by estimating the effective population size trough time (expressed as the product of *N*_e_ and generation τ) by using the Bayesian skyline plot reconstruction as implemented in the TRACER program.

### Phylogenetic reconstruction and geographic population structure

Aligned sequences were first tested for the presence of recombination using the genetic algorithm for recombination detection (GARD) approach (Kosakovsky Pond et al. 2006) as implemented in the HyPhy software package (Kosakovsky Pond et al. 2005). In short, GARD estimates topological incongruence by searching the space of all possible locations for recombination break-points in the alignment and inferring phylogenies for each putative nonrecombinant fragment. The phylogenetic relationships were reconstructed with two different approaches. A Bayesian-based maximum clade credibility (MCC) tree was inferred by the BEAST analyses as described above and a maximum likelihood (ML) tree was reconstructed using MEGA 5.05 (Tamura et al. 2011) and the implemented HKY85 substitution model (Hasegawa et al. 1985) with gamma correction for site rate.

Clustering of the European CHV-1 subtype I sequences from the eight populations sampled recently was also conducted on the amino acid level using a principal component analysis (PCA). The method is implemented in JALVIEW (Waterhouse et al. 2009) and generates the components by an eigenvector decomposition of the matrix formed from the sum of BLOSUM scores at each aligned position between each pair of sequences.

To further assess the geographic structure of CHV-1 subtype I, we used spatial analyses of molecular variance based on *F*-statistics as implemented in SAMOVA (Dupanloup et al. 2002). The program performs a simulated annealing procedure that aims at maximizing among group variance (*F*_CT_) and minimizing among populations within group variance (*F*_SC_). The goal is to define groups of populations that are geographically homogeneous and genetically maximally differentiated from each other. To avoid spurious results due to initial population configuration, 100 initial conditions were used as recommended by Dupanloup et al. (2002).

Finally, a Mantel test with 1000 iterations was performed to determine the significance of isolation-by-distance (IBD) between populations using the Isolation-by-Distance Web Service 3.21 (Jensen et al. 2005). As suggested by Slatkin (1993), correlation was tested between matrices of linearized *F*_ST_ values and the logarithm of geographic distances.

## Results

### Genetic diversity of virus and fungus populations

Table 2 displays the indices of genetic diversity in the eight virus and fungus populations. After rarefaction analysis, mean (±SD) richness of haplotypes in the virus populations was 15.25 (±1.49), mean diversity of haplotypes was 2.67 (±0.11) and mean nucleotide diversity was 0.00709 (±0.00249). No significant differences (*P* > 0.05) in either of these indices were observed between the populations from the central range and the populations from the southern range. While a few haplotypes occurred more than once within populations there were no shared haplotypes between populations. In the fungal populations, highly significant (*P* ≤ 0.001) differences existed between the populations from the central and the populations from the southern range. Diversity and richness of vc types (see also Table S1) were substantially higher in the populations from the central range. The mean richness of vc types was 7.75 (±7.31) and mean diversity of vc types was 1.05 (±1.13).

**Table 2:** Indices of genetic diversity in virus and fungus populations

		Virus						Fungus				
			
Name	Range	N_Virus_	R_Haplo_	R_Haplo[17]_^1^	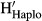	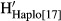 2	π	N_Fungus_	R_Vc_	R_Vc[44]_^3^	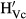	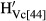 4
Go	Central	50	43	16	3.71	2.75	0.00820	97	14	10	1.91	1.63
Pu	Central	42	38	16	3.61	2.83	0.01183	97	24	17	2.57	2.54
Ka	Central	38	27	14	3.12	2.56	0.00488	46	17	17	2.37	2.26
Iv	Central	30	25	15	3.14	2.64	0.00516	44	13	13	1.87	1.87
Ra	Southern	55	51	17	3.91	2.75	0.00713	72	2	2	0.13	0.11
He	Southern	41	29	13	3.00	2.51	0.00445	93	1	1	0.00	0.00
Bu	Southern	33	32	17	3.45	2.75	0.00616	96	1	1	0.00	0.00
Ya	Southern	17	14	14	2.59	2.59	0.00893	89	1	1	0.00	0.00

1 Haplotype richness after rarefaction analysis for the smallest sample size (*n* = 17).

2 Shannon–Wiener's index for haplotype diversity after rarefaction analysis for the smallest sample size (*n* = 17).

3 Vc type richness after rarefaction analysis for the smallest sample size (*n* = 44).

4 Shannon–Wiener's index for vc type diversity after rarefaction analysis for the smallest sample size (*n* = 44).

### Phylogenetic tree and geographic clustering

**Figure 2:**
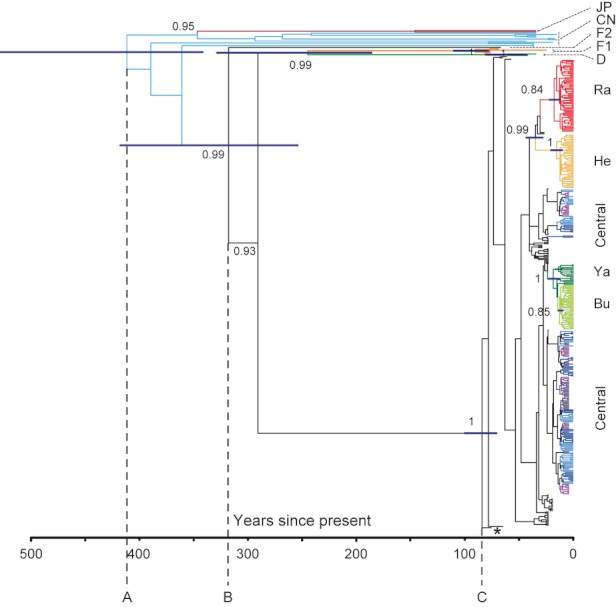
Bayesian-based coalescent phylogeny (maximum clade credibility tree) of *Cryphonectria hypovirus 1* (CHV-1)*;* deep phylogeny including samples from the European CHV-1 subtypes I, F1, F2 and D as well as samples from China and Japan. Node-bars are time estimates (95% highest posterior density) and values at nodes are posterior probabilities for the main phylogenetic groups. The bottom axis represents time estimates (years since present). Node A is the root of the tree, node B represents the time to the most recent common ancestor (TMRCA) for the European subtypes, and node C is the TMRCA for the European subtype CHV-1 subtype I. The asterisk indicates the position of the reference sequence CHV-1/Euro7. The populations Go (light blue), Pu (dark blue), Ka (pink), and Iv (purple) represent the central populations from Switzerland and Bosnia-Herzegovina, and the populations Ra (red), He (orange), Bu (light green), and Ya (dark green) represent the southern populations from the southern Balkans and Turkey. The color code is the same as in the Figures 3 and 4.

According to Bayes factor comparisons, the lognormal relaxed clock model scored significantly higher than the strict clock model (*K* = 100.22), all else being equal. Similarly, the Bayesian skyline plot was selected over the constant size population model (*K* = 143.90) and over the exponential growth model (*K* = 43.68). The GARD approach did not detect any recombination break-points that significantly affected the topologies of phylogenetic reconstructions. Deep phylogenetic relationships among CHV-1 isolates were reconstructed by using both the MCC tree obtained from the BEAST analysis (Fig. 2) and the ML tree obtained from the MEGA analysis (Fig. S1). Both trees inferred the same pattern. The isolates from China and Japan were all located at the base of the tree, supporting the Asian origin of the virus. The European CHV-1 isolates formed a distinct cluster divergent from the Asian isolates. All CHV-1 subtype I isolates formed a maximally supported monophyletic clade in both the MCC (1.0 posterior probability) and ML (100% bootstrap values) analyses. Isolates from the recently sampled four central populations Go, Pu, Ka, and Iv were intermixed. Consequently, none of these populations had significant posterior probabilities, suggesting that they do not represent distinct clades (Fig. 2). In contrast, each of the four southern populations Ra, He, Bu, and Ya represented a distinct cluster supported by high posterior probabilities. Furthermore, Ra and He diverged at a different node than Bu and Ya from the central populations.

### Nucleotide substitution rate and time to the most recent common ancestor

**Figure 3:**
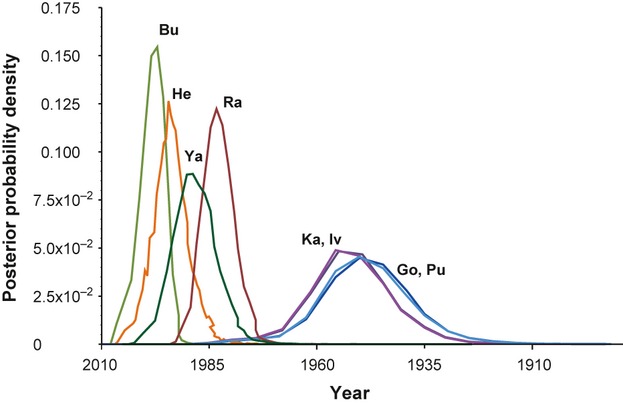
Bayesian posterior density plot of the time of most recent common ancestors for the eight European *Cryphonectria hypovirus 1* subtype I populations in this study. The populations Go, Pu, Ka, and Iv represent the central populations from Switzerland and Bosnia-Herzegovina, and the populations Ra, He, Bu, and Ya represent the southern populations from the southern Balkans and Turkey. The color code is the same as in the Figures 2 and 4.

The Bayesian estimates gave a mean evolutionary rate of 3.7 × 10^−4^ (lower 95% highest posterior density [HPD] 2.4 × 10^−4^, upper 95% HPD 6.1 × 10^−4^) substitutions/site/year. Given this evolutionary rate, the estimated TMRCA for the four European subtypes I, F1, F2, and D of CHV-1 was 322 (95% HPD 411 – 251) years (node B in Fig. 2). The TMRCA for the dominant European subtype CHV-1 subtype I was 88 (95% HPD 102 – 72) years (node C in Fig. 2). Within the eight European populations of CHV-1 subtype I, the TMRCA estimates supported the distinction of five genetic lineages (Fig. 3) as also suggested by the phylogenetic trees. The four populations forming the central population cluster had very similar TMRCA estimates with values of 64 years (1946) for both Swiss populations (Go, Pu) and 61 years (1949) for both Bosnian populations (Ka, Iv). Estimates for the four southern populations were clearly more recent, i.e. 28 years (1982) for Ra, 22 years (1988) for Ya, 15 years for He (1995), and 12 years (1998) for Bu. Although the TMRCA estimates of the southern populations were partially overlapping, the posterior probabilities of TMRCA support the genetic and geographic clustering results of one undifferentiated central population and four distinct southern populations described above.

The reconstruction of the demographic history of CHV-1 is depicted in Figure S2. The skyline plot suggested that the effective population size of the virus increased in distinct periods of growth (G) and stasis (S) and not continuously.

### Principal component analysis of population differentiation

**Figure 4:**
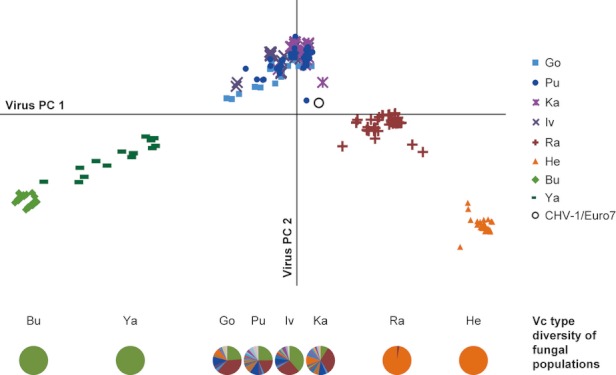
Principal component analysis (PCA) of amino acid differentiation among isolates of the eight *Cryphonectria hypovirus 1* subtype I populations using the sum of pairwise BLOSUM scores for eigenvector decomposition; Bu and Ya from Turkey, Go and Pu from Switzerland, Iv and Ka from Bosnia-Herzegovina, Ra from Macedonia, and He from Greece. The color code is the same as in the Figures 2 and 3. The sequence of *Cryphonectria hypovirus 1*/Euro7 from Italy was added as a reference. Pie graphs displaying the fungal vegetative compatibility (vc) type diversity in these eight populations were aligned to the PCA graph.

In agreement with the phylogenetic analysis and the TMRCA estimates, the PCA of amino acid sequence differentiation among European CHV-1 subtype I isolates suggested the presence of one large undivided population cluster consisting of the four populations Go, Pu, Ka, Iv referred to as the central populations and of four individual populations that differentiate from the central cluster in two directions (Bu and Ya vs. Ra and He) (Fig. 4). In Fig. 4, the PCA of the CHV-1 subtype I amino acid sequence differentiation was contrasted against the vc type diversity of the respective fungal populations. The vc diversity was high in all central populations, while there was only one dominant vc type in each of the southern populations. Furthermore, the populations Bu and Ya were dominated by a different vc type than the populations Ra and He.

### Geographic population structure

The SAMOVA result was congruent with the population clustering detected in the phylogenetic trees and the PCA. The best partitioning of the genetic diversity by SAMOVA was obtained when populations were divided into *K = 5* geographic groups (*F*_CT_ = 0.4759, *P* = 0.014; Table 3). The four central populations (Go, Pu, Ka, and Iv) fell within a single cluster, although the geographic distance between them was up to 918 km (Table S2). The four southern populations (Bu, Ya, Ra, and He), on the other hand, were each inferred as separate groups.

**Table 3:** Spatial analysis of molecular variance (SAMOVA) for eight European populations of *Cryphonectria hypovirus 1* subtype I

Source of variation	df	Sum of squares	Variance components	Percentage of variation (%)	*P*
*K* = 2 (Go, Pu, Ka, Iv, Bu, Ya, Ra) versus (He)					
Among groups	1	201.379	1.610	26.25	0.109
Among populations within group	6	474.900	2.059	33.57	<0.001
Within populations	298	734.668	2.465	40.19	<0.001
*K* = 3 (Go, Pu, Ka, Iv, Ra) versus (He) versus (Bu, Ya)					
Among groups	2	449.551	2.581	41.90	0.006
Among populations within group	5	226.729	1.113	18.07	<0.001
Within populations	298	734.668	2.465	40.03	<0.001
*K* = 4 (Go, Pu, Ka, Iv) versus (He) versus (Ra) versus (Bu, Ya)					
Among groups	3	584.940	2.547	45.57	<0.001
Among populations within group	4	91.340	0.577	10.33	<0.001
Within populations	298	734.668	2.465	44.11	<0.001
*K* = 5 (Go, Pu, Ka, Iv) versus (He) versus (Ra) versus (Bu) versus (Ya)					
Among groups	4	614.387	2.655	47.59	0.014
Among populations within group	3	61.892	0.459	8.23	<0.001
Within populations	2	734.668	2.465	44.18	<0.001

Abbreviations are the same as the population abbreviations in Table 1.

The Mantel test for the pairwise correlation of linearized *F*_ST_ calculated from molecular distances of pairwise haplotype differences and geographic distances was not significant (Fig. S3; *r* = 0.0113, *P* = 0.52), suggesting no IBD among the European populations of CHV-1 subtype I.

## Discussion

Evolutionary congruence between parasite and host on the spatial scale is expected, if the parasite depends on the host for dispersal (Holmes 2004, 2008). This is largely the case for the fungal virus CHV-1 and has also been observed in other RNA viruses (Real et al. 2005; Biek et al. 2007; Nadin-Davis et al. 2010; Torres-Pérez et al. 2011). Ecological and demographic factors that influence the spread of the host have therefore also a great impact on the dispersal and evolution of the virus. In this study, we conducted a large-scale sampling across Europe, analyzed the population structure of the fungal virus CHV-1 and reconstructed its phylogenetic history using a coalescent approach. Our results strongly suggest that the evolution of CHV-1 is spatially, but not temporally, congruent with the evolution of its host *C. parasitica*. This is the first investigation of CHV-1 genome sequences obtained from a wide area in Europe. Furthermore, it is the first study in the *Cryphonectria*-hypovirus pathosystem that allowed the estimation of the dates of introduction and population emergence based on genetic sequence information.

The phylogenetic analyses performed in our study support an Asian origin of the European CHV-1 isolates. Previous analyses (Liu et al. 2007) of Asian and European CHV-1 discovered that the genetic diversity among Asian isolates was higher than among European isolates, indicating that the European CHV-1 populations had emerged more recently. The four European subtypes of CHV-1 (I, F1, F2, and D) were more closely related to each other than to any of the Asian isolates. Estimates of the TMRCA for the four European CHV-1 subtypes indicated that the subtypes diverged more than 300 years ago, i.e. prior to the first records of *C. parasitica* in Europe. The finding that the emergence of the subtypes predates the introduction of *C. parasitica* to Europe is in agreement with the results of a previous study by Gobbin et al. (2003) and suggests that multiple introductions of CHV-1 to Europe have occurred.

As expected, all isolates from the eight recently sampled European populations mapped to the CHV-1 subtype I cluster and formed a well-supported monophyletic clade. We detected five distinct viral lineages within this clade. Our results suggested the existence of one large undivided population cluster of CHV-1 subtype I and four individual small clusters. This cluster included the two populations sampled in southern Switzerland (Go and Pu) and the two populations sampled in north-western Bosnia-Herzegovina (Ka and Iv), and thus extended over a wide geographic area. These populations were referred to as the central populations, in accordance with the denomination used in a previous study on the fungal host populations in these regions (Milgroom et al. 2008). The populations from Macedonia (Ra) and Greece (He) and the two populations from Turkey (Bu and Ya), on the other hand, were inferred as four distinct southern populations that diverged from the central populations more recently at two different nodes. It is remarkable that the Swiss and the Bosnian populations form a homogenous population cluster, even though the geographic distance between them is large (approximately 800 km). The geographic distances between the populations Ra and He (approximately 300 km) and between Bu and Ya (approximately 30 km) are much shorter and, nevertheless, they each represent a distinct phylogenetic group. The lack of significant IBD among the CHV-1 populations suggested that the emergence of CHV-1 was not shaped by a continuous dispersal from the central range toward the southern range.

All analyses performed in this study, i.e. the phylogenetic analyses using a Bayesian-based coalescent approach and a ML approach, estimates of the TMRCA, the PCA of amino acid differentiation and the SAMOVA, inferred the same pattern for the evolution of the CHV-1 subtype I populations. According to the TMRCA estimates, the populations from Switzerland were the oldest among the eight CHV-1 subtype I populations investigated in this study, but only a few years older than the populations from Bosnia-Herzegovina. The estimated date of TMRCA of the Swiss populations coincides with the first report of *C. parasitica* in Switzerland in 1948 (Robin and Heiniger 2001), indicating that there was no or only a short time lag between the introduction of the fungus and the virus. This has already been suggested by Heiniger and Rigling (1994). Some reports, however, indicated an absence of CHV-1 at the front of *C. parasitica* colonization in Europe (reviewed in Robin and Heiniger 2001). Our results suggest that the presence of CHV-1 may have remained undetected for several years, as CHV-1 was not identified in Switzerland before 1975. *C. parasitica* was first found in Europe in northern Italy in 1938 and the first signs of virus infection were already observed in 1951 (Biraghi 1953). Alternatively, the fungal individuals at the forefront of population expansion and colonization could have been virus-free. The transmission rate of CHV-1 into the asexual spores of *C. parasitica* is <100% (Prospero et al. 2006). Virus-infected *C. parasitica*, therefore, produce some virus-free offspring that grow faster, sporulate more, and thus, disperse more rapidly to new regions than their virus-free progenitors. The spread of the virus may therefore have lagged shortly behind the spread of the fungus. If CHV-1 was introduced to Italy together with *C. parasitica*, as our findings indicate, it colonized the central range in spreading north to southern Switzerland and east through the neighboring countries Slovenia and Croatia to north-western Bosnia-Herzegovina.

**Figure 5:**
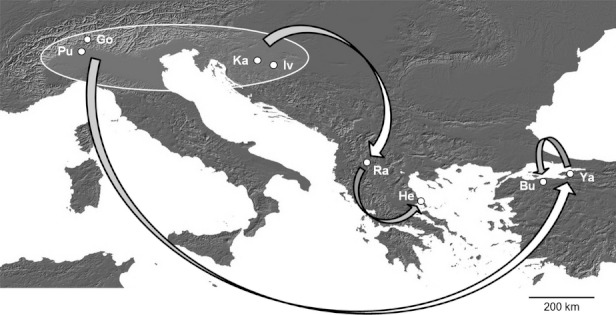
Graphical illustration of the evolutionary scenario inferred for *Cryphonectria hypovirus 1* subtype I in Europe. The framed area comprises the central populations. Arrows indicate waves of introductions toward the southern range. The locations of the eight populations sampled in this study (abbreviations as in Table 1) are indicated by white dots.

Estimates of the TMRCA and the skyline plot suggested that CHV-1 rapidly established in the central range (1946–1949). However, only after a long time period of stasis (approximately 35 years) during which the effective number of viruses did not change significantly a first wave of invasion from the central to the southern range occurred. During this time period (1980–1990), the populations Ra (southern Balkans) and Ya (Turkey) were founded almost simultaneously by two independent invasions and the effective number of viruses increased rapidly. After the establishment of these populations, the effective number of viruses reached a plateau until a second wave of invasion was initiated. Again roughly during the same time period (1995–2000), the populations He (southern Balkans) and Bu (Turkey) were founded by invasions from geographically proximate populations (represented in our study by Ra and Ya, respectively). Figure 5 summarizes the evolutionary scenario proposed for CHV-1.

The skyline plot indicates a steep drop in population size during a short period at around the time of the second wave of invasion. This could be a sign for population bottlenecks due to new founding events. The bottleneck was followed by an extended period of population growth, which allowed the southern CHV-1 populations to quickly recover from the genetic bottlenecks. In fact, we observed a high genetic diversity in all CHV-1 populations investigated, in the central as well as in the southern populations, which further supports this assumption. A rapid population growth and increase in diversity after genetic bottlenecks have also been observed in other viruses such as in tomato yellow leaf curl China virus (Ge et al. 2007) and human immunodeficiency virus (Kouyos et al. 2011; Ragonnet-Cronin et al. 2012). This contrasts with the situation in the populations of the fungal host *C. parasitica*. The clonal population structures in the southern range (evident from the presence of only one dominant vc type) reflect strong genetic bottlenecks in *C. parasitica* resulting from recent founder events. The reason for the incongruence in genetic diversity between the fungal and the viral populations may be differences in their evolutionary rate. We estimated a mean nucleotide substitution per site per year of 3.7 × 10^−4^ for CHV-1. No information is available on nucleotide substitution rates for closely related virus species, but the rate estimated falls in the range of 10^–2^ to 10^–5^ reported for RNA viruses (Duffy et al. 2008 and references therein). This rate, however, exceeds the evolutionary rate of approximately 10^−9^ reported for ascomycete fungi (Kasuga et al. 2002) such as *C. parasitica* by several orders of magnitude. Our observation agrees with other observations of virus–host coevolution. It was described previously that, on the temporal scale, the evolution of RNA viruses is usually not congruent with the evolution of their hosts, due to differences in nucleotide substitution rates (Holmes 2004, 2008).

On the spatial scale, the evolutionary scenario inferred for CHV-1 in our study matches the scenario inferred for the *C. parasitica* populations. The first records of *C. parasitica* in Europe suggested that it was introduced on chestnut wood or plants through the port of Genoa in northern Italy around 1938 (Heiniger and Rigling 1994). After establishment, the *C. parasitica* populations most likely expanded from northern Italy to the nearby areas (Milgroom et al. 2008; Dutech et al. 2010; Jezic et al. 2012; Prospero and Rigling 2012). Milgroom et al. (2008) characterized *C. parasitica* population structures from the central and the southern range in Europe based on vc types and sequence characterized amplified region haplotypes. The study revealed a high genetic diversity and very similar structures of the fungal populations in the central range. The populations in the southern range, however, were mainly clonal. Different clones dominated the *C. parasitica* populations in the southern Balkans and the populations in Turkey, in accordance with our observations. Studies from Slovenia (Krstin et al. 2011), Croatia (Krstin et al. 2008; Jezic et al. 2012), and Bosnia-Herzegovina (Trestic et al. 2001) further suggested that the division between the genetically diverse central populations and the clonal southern populations runs through Bosnia-Herzegovina. The *C. parasitica* populations in Slovenia, Croatia, and north-western Bosnia-Herzegovina shared the characteristics with the other central populations. The *C. parasitica* populations in southern Bosnia-Herzegovina, however, were dominated by the same clone as the populations in the southern Balkans. Milgroom et al. (2008) therefore concluded that the clonal population structures of the fungus in the southern Balkans and in Turkey were the results of two independent invasion events.

The congruence between the invasion histories inferred for *C. parasitica* and for CHV-1 is striking. As explained earlier, the spread of CHV-1 is completely host-dependent. CHV-1 disperses in virus-infected mycelial particles or in virus-infected asexual spores (conidia) of the fungus over very short distances. The dispersal of CHV-1 is therefore linked to ecological factors influencing the spread of the fungal host. It was shown in other RNA viruses such as rabies virus of fox (Real et al. 2005), of raccoon (Biek et al. 2007) and of bat (Nadin-Davis et al. 2010) and in Andes virus of rats (Torres-Pérez et al. 2011) that their phylogenetic history was tightly connected to host demography and geographic barriers limiting host movement and contacts. CHV-1 is not transmitted to the sexual spores (ascospores) of the fungus (Carbone et al. 2004), which are also transported by wind, and thus would enable the spread of fungal propagules over longer distances. The data available on *C. parasitica* to date suggest that vector-aided transport of virus-infected mycelium or virus-infected conidia of *C. parasitica* was responsible for the long-distance dispersal of both the fungus and the virus. An important vector may, thus, have been infected chestnut plants, timber or firewood (Prospero et al. 2006) traded and transported across Europe.

The rapid colonization of the central range by CHV-1 may be explained by the rapid expansion of the fungal population across the forests on the south facing slopes of the Alps in Switzerland, Italy, Slovenia, and Croatia and on the north-east facing slopes of the Dinaric Alps in north-western Bosnia-Herzegovina. The geography and the relatively continuous forest belt in the central range with widespread occurrence of chestnut might have been favorable for the short-distance dispersal of (virus-infected) mycelial fragments and conidia. In Bosnia-Herzegovina, however, the Dinaric Alps, which rise well above the timber line, cross the country from west to east, and thus impose a major barrier for the southward spread of the fungus and virus. The spread of virus-infected *C. parasitica* would therefore have to occur over long distances, for example, by infected plant material transported by humans. This is consistent with the observed division between the genetically diverse populations of *C. parasitica* in north-western Bosnia-Herzegovina and the clonal populations in southern Bosnia-Herzegovina (Trestic et al. 2001). As discussed by Biek and Real (2010), spatial heterogeneity affects the population dynamics of the host as well as that of a host-dependent parasite. The quarantine measures that were taken after the breakout of the European chestnut blight epidemic, however, may have been effective in containing *C. parasitica* (and also CHV-1) within the central range for many years. According to our TMRCA estimates, the Macedonian CHV-1 population investigated in this study was founded in the early 1980s. Interestingly, this falls within the time of political instability in the state of former Yugoslavia after the death of president Tito in 1980. At that time, civil commotions increased and Yugoslavia started to fall apart, which may have been associated with increased movements of people and material across the state. In 1991, Macedonia and Bosnia-Herzegovina, which belonged to former Yugoslavia, became independent. Subsequently, a dispute arose between Greece and Macedonia about the name and flag of the new Macedonian republic, which culminated in a trade embargo against Macedonia. The embargo was relieved in 1995 and trade between the independent Macedonian republic and Greece increased. It is striking that 1995 is also the TMRCA estimate of the CHV-1 population in Greece. These coincidences of political events and the estimated emergence of new CHV-1 populations may be accidental. However, it is likely that the political situation in the southern Balkans at the end of the 20th century facilitated long-distance dispersal of *C. parasitica* and CHV-1 due to alterations in the volume and direction of fluxes of trade and travel across the region.

Similarly, political tensions between Greece and Turkey and restrictions in trade between these two countries at the time may explain why Greece was (despite geographic proximity) clearly not the source for the CHV-1 and *C. parasitica* populations in Turkey. Turkey was most likely invaded by CHV-1 and *C. parasitica* on diseased plant material that had been shipped from the central range. The lack of genetic differentiation among the central populations does not allow to pin-point the exact source of the Turkish populations. However, one likely source would be Italy, the European centre of chestnut production and breeding (Castellini et al. 2009). Shipment of high quality chestnut germplasm from Italy to other European countries has not been uncommon. The hypothesis that CHV-1 invaded Turkey by boat is supported by the TMRCA estimates. The population Ya, which is closer to the port of Istanbul, emerged 10 years prior to the population Bu, which is located further west. Forests are patchy in this region and the two populations are not connected by a continuous forest belt. Hence, the population Bu was probably founded by a rare long-distance dispersal event that explains the time-lag between the emergence of the two populations as well as the persistent population differentiation.

An alternative explanation for the spatial population structure could also be simple stochastic long dispersal events during the geographic expansion. Recurrent founding events during a gradual diffusion of the virus combined with a slow expansion in the newly invaded areas could also have yielded the genetic pattern observed. Additional sampling in the uncovered geographic areas would be needed to clearly reject this alternative hypothesis.

*Cryphonectria hypovirus 1* plays an important role as a biocontrol agent of chestnut blight in Europe. In extensively managed or natural ecosystems, such as forests, the long-term establishment of the biocontrol agent is desirable (Payne et al. 1988). Ideally, a biocontrol agent is spreading independently and persists in the ecosystem, exerting a continuous control. The spatial and temporal evolution of biocontrol agents is therefore crucial for the sustainability and effectiveness of the disease control. However, little is known about these evolutionary processes in natural biocontrol systems (Hufbauer and Roderick 2005). CHV-1 occurred naturally in *C. parasitica* populations and spread independently across Europe (Heiniger and Rigling 1994). In our study, CHV-1 was prevalent in all *C. parasitica* populations investigated. Our results indicate that CHV-1 successfully invaded newly established populations of the destructive pathogen *C. parasitica*, and thus probably contributed to prevent the development of devastating chestnut blight epidemics, such as observed in North America (Anagnostakis 1982; Milgroom and Cortesi 2004). Furthermore, our study showed that the newly founded CHV-1 populations had quickly overcome genetic bottlenecks and reached a high genetic diversity due to the rapid evolutionary rate of CHV-1. The results of another study conducted in the same populations revealed that variation in CHV-1 virulence toward *C. parasitica* was present in all populations, and that there was no evidence for reduced disease control in either the older or the newly founded populations (Bryner and Rigling 2012). In conjunction, these results are promising for the preservation of the European chestnut forests. Studies investigating the spatial and temporal evolution of biocontrol agents in other ecosystems would be useful to define the characteristics and requirements of successful and sustainable biocontrol agents in general. Our study indicates that CHV-1 has characteristics that are ideal for a sustainable disease control in (semi-) natural ecosystems.

In conclusion, our analysis of genome sequences obtained from CHV-1 populations across Europe suggests that the evolution of CHV-1 was spatially congruent with the evolution of its host *C. parasitica*. We were able to show that CHV-1 had most likely been introduced together with *C. parasitica* and that its spread across Europe was not continuous. The use of a coalescent approach allowed us to estimate the dates of population emergence in the different European regions and to reconstruct the invasion history of CHV-1. We discussed the consistence of geographic, vegetation-related, demographic, economic, and political factors with the correlated invasion pattern inferred for CHV-1 and *C. parasitica* in Europe.

## Data Archiving Statement

The CHV-1 subtype I sequences from the eight European populations sampled in this study are deposited under the Genbank accession numbers JX969839 to JX970144.

## Supporting Information

**Figure S1**. Maximum-likelihood tree of phylogenetic relationships of *Cryphonectria hypovirus 1* (CHV-1); deep phylogeny including samples from the European CHV-1 subtypes I, F1, F2, and D as well as samples from China and Japan. Branch lengths are drawn to scale, with the scale bar indicating the number of nucleotide substitutions per site. Bootstrap support values >80% are indicated on branches.

**Figure S2**. Effective population size (*N*_e_*τ) change of the eight European *Cryphonectria hypovirus 1* subtype I populations analyzed in this study represented as a Bayesian skyline plot through time. The gray area represents 95% highest posterior density (HPD) intervals. Dotted lines are estimates of the time to the most recent common ancestor (TMRCA and 95% HPD). Arrows indicate periods of growth (G) and stasis (S), respectively.

**Figure S3**. Genetic distance (linearized *F*_ST_) is plotted against the logarithm of geographic distances (in km). No isolation by distance was inferred from matrix correlation between genetic distance (linearized *F*_ST_) and log geographic distance (km). Line is the reduced major axis (RMA) regression. The correlation between genetic distance and geographic distance was not significant (*Z* = 99.6662, *r* = 0.2378, *P* = 0.94) based on 1000 randomizations using Mantel test.

**Table S1.** Vegetative compatibility (vc) types observed in the eight populations of *Cryphonectria parasitica*.

**Table S2.** Pairwise values for linearized *F*_ST_ (above diagonal) and geographic distance (below diagonal) used in the Mantel test for significance of isolation-by-distance (IBD) between populations of *Cryphonectria hypovirus 1* subtype I.
