# Supplementary figures and images for: Invasion history and demographic pattern of Cryphonectria hypovirus 1 across European populations of the chestnut blight fungus
Source: Ecol Evol. 2012 Nov 22;2(12):3227–41. doi: 10.1002/ece3.429 (PMC3539014; doi:10.1002/ece3.429)

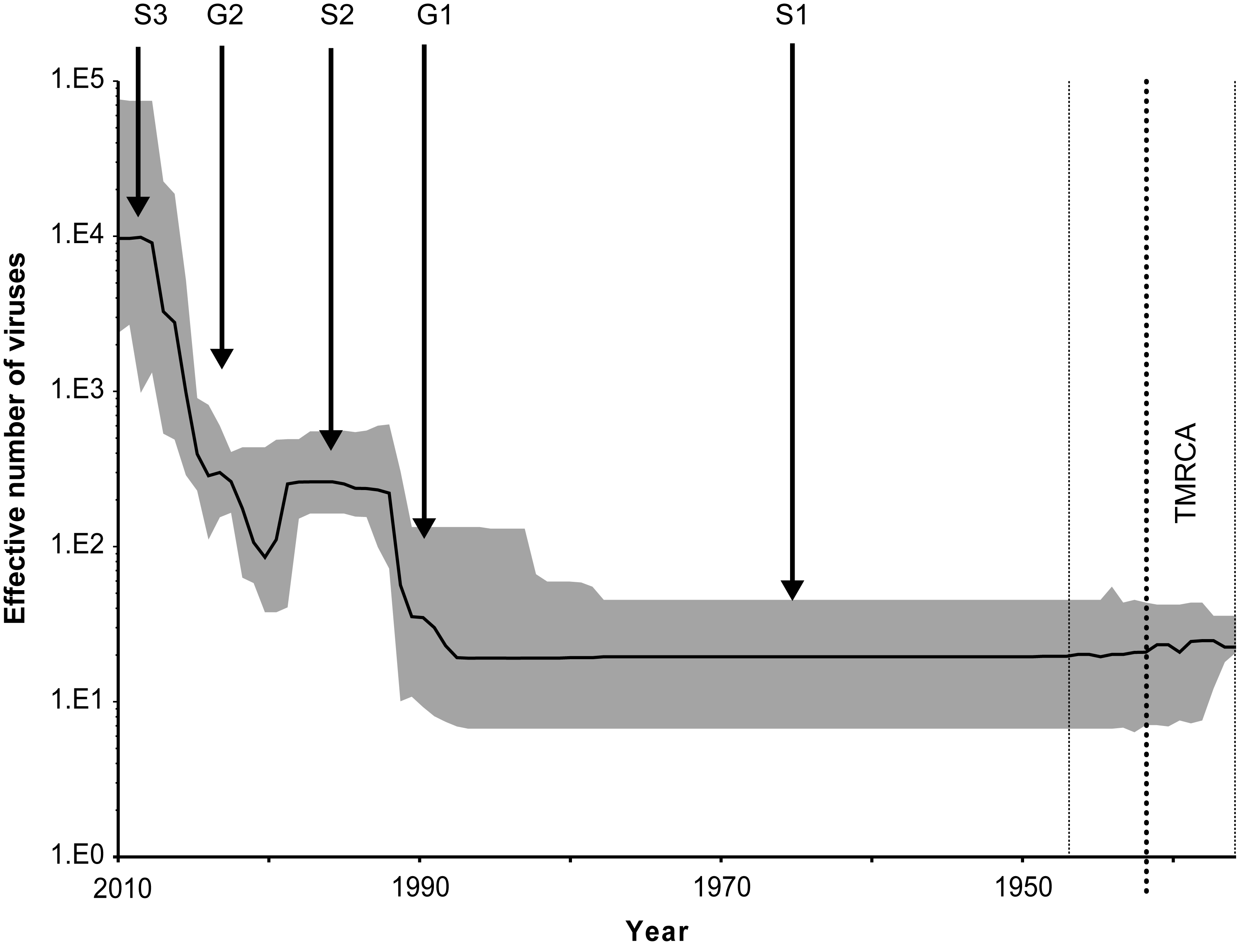

Supplement: Supplementary file 2 [file ece30002-3227-SD2.tif]
