# Supplementary material for: Invasion history and demographic pattern of Cryphonectria hypovirus 1 across European populations of the chestnut blight fungus
Source: Ecol Evol. 2012 Nov 22;2(12):3227–41. doi: 10.1002/ece3.429 (PMC3539014; doi:10.1002/ece3.429)
Supplement: Supplementary file 4 [file ece30002-3227-SD4.doc]

**Supporting information**


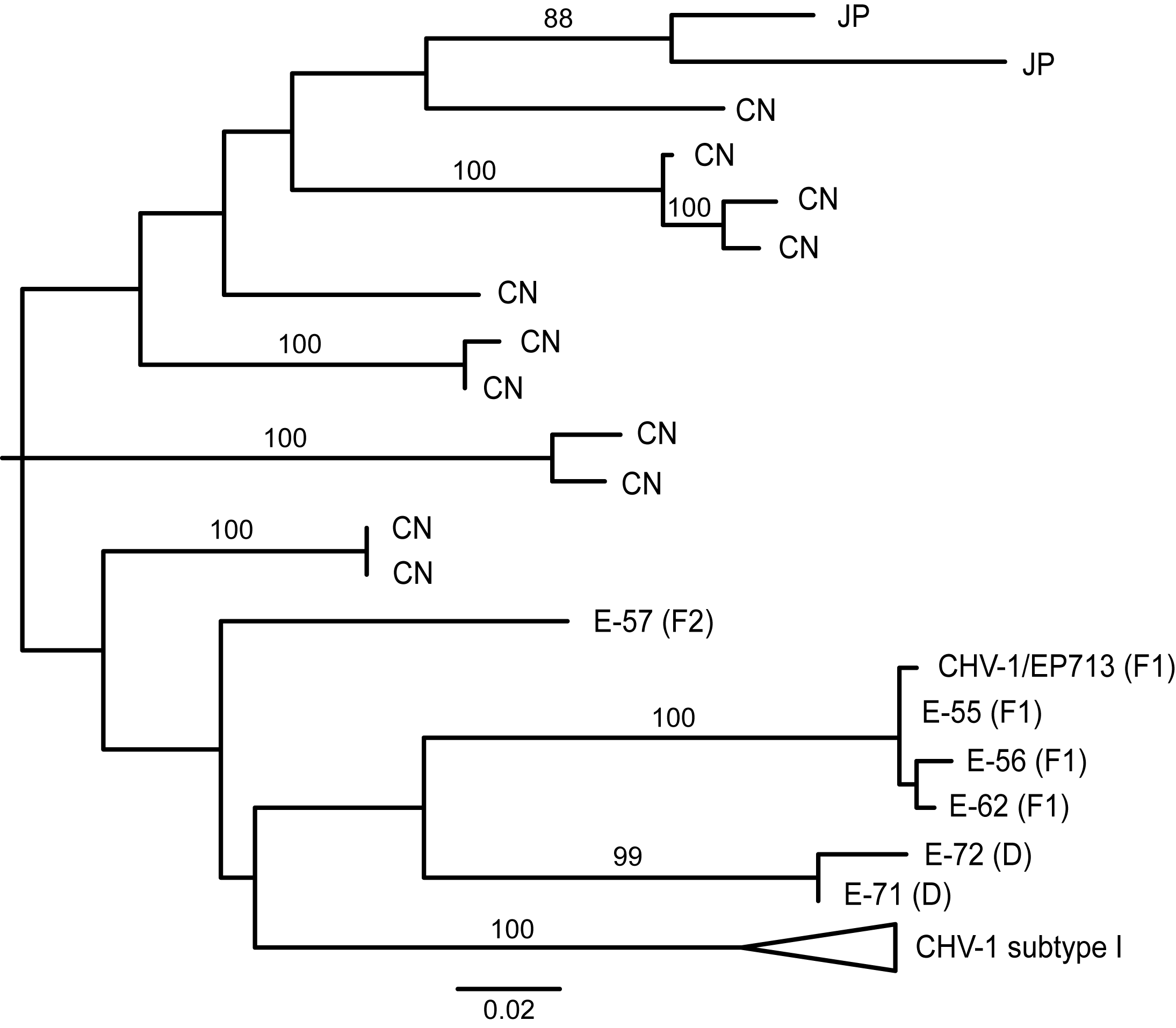


**Figure S1** Maximum-likelihood tree of phylogenetic relationships of *Cryphonectria hypovirus 1* (CHV-1); deep phylogeny including samples from the European CHV-1 subtypes I, F1, F2 and D as well as samples from China and Japan. Branch lengths are drawn to scale, with the scale bar indicating the number of nucleotide substitutions per site. Bootstrap support values >80% are indicated on branches.


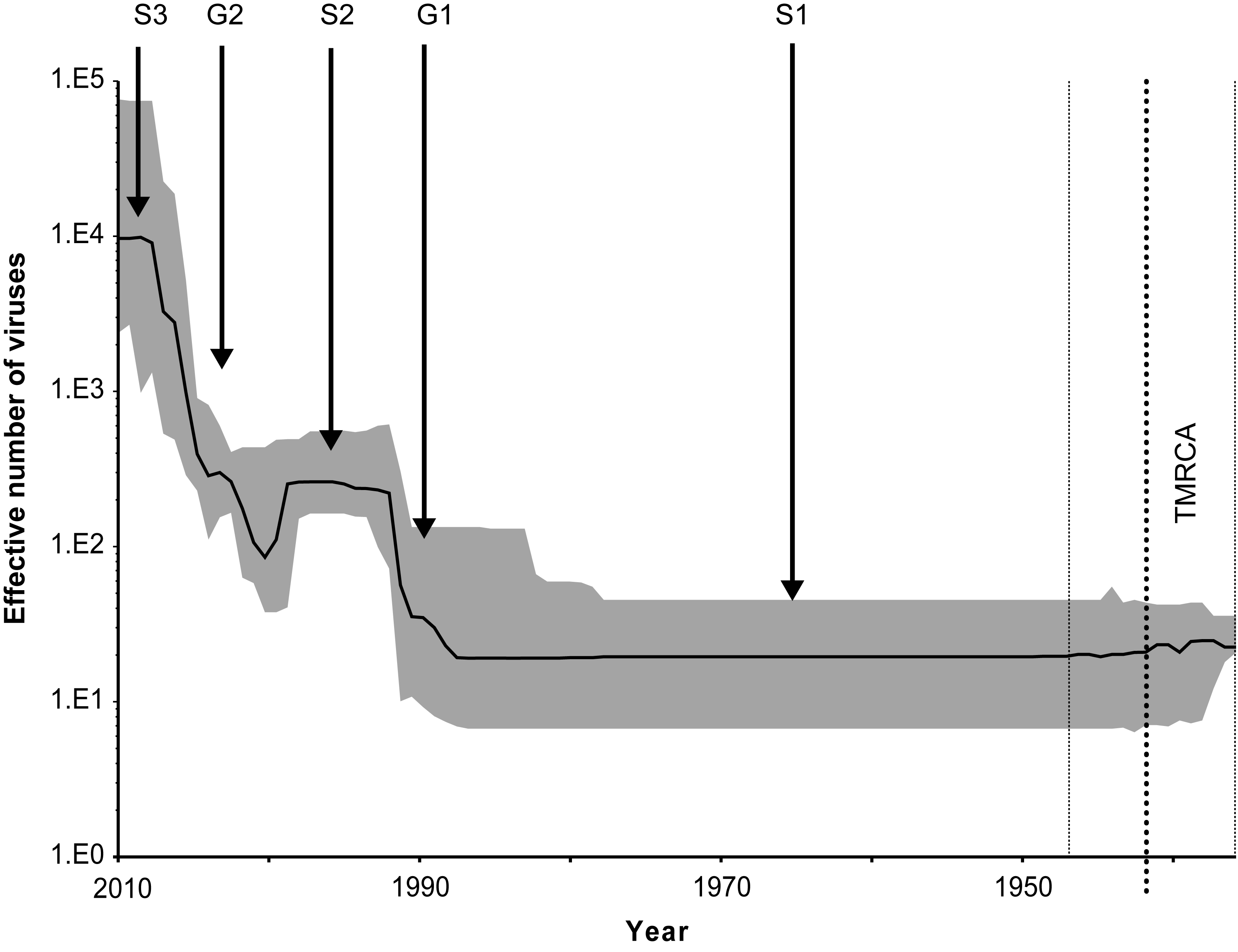


**Figure S2** Effective population size (N_e_*τ) change of the eight European *Cryphonectria hypovirus 1* subtype I populations analyzed in this study represented as a Bayesian skyline plot through time. The grey area represents 95% highest posterior density (HPD) intervals. Dotted lines are estimates of the time to the most recent common ancestor (TMRCA and 95% HPD). Arrows indicate periods of growth (G) and stasis (S), respectively.


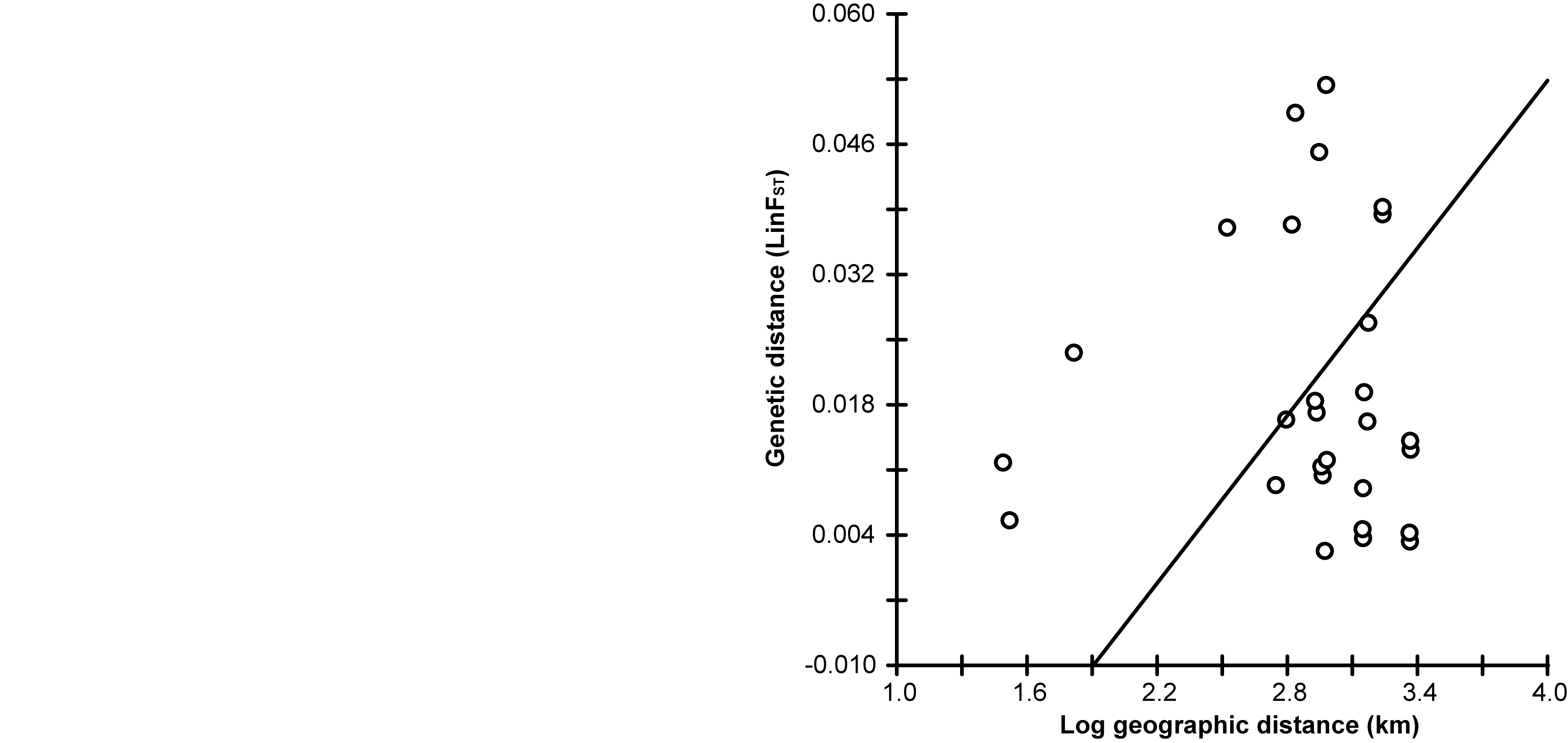


**Figure S3** Genetic distance (linearized F_ST_) is plotted against the logarithm of geographic distances (in km). No isolation by distance was inferred from matrix correlation between genetic distance (linearized F_ST_) and log geographic distance (km). Line is the reduced major axis (RMA) regression. The correlation between genetic distance and geographic distance was not significant (Z = 99.6662, r = 0.2378, P = 0.94) based on 1000 randomizations using Mantel test.

**Table S1** Vegetative compatibility (vc) types observed in the eight populations of *Cryphonectria parasitica*.

| Vc Type | Go | Pu | Ka | Iv | Ra | He | Bu | Ya |
| --- | --- | --- | --- | --- | --- | --- | --- | --- |
| EU-1 | 23 | 24 | 4 | 17 |  |  | 96 | 89 |
| EU-2 | 37 | 18 | 15 | 12 | 2 |  |  |  |
| EU-3 | 3 | 2 | 1 | 1 |  |  |  |  |
| EU-4 | 4 | 4 |  | 1 |  |  |  |  |
| EU-5 | 10 | 10 | 4 | 3 |  |  |  |  |
| EU-6 | 4 | 8 | 3 |  |  |  |  |  |
| EU-7 |  | 2 | 2 | 2 |  |  |  |  |
| EU-8 |  | 2 | 1 | 1 |  |  |  |  |
| EU-9 |  | 1 | 1 |  |  |  |  |  |
| EU-10 |  |  | 1 |  |  |  |  |  |
| EU-11 |  | 1 |  |  |  |  |  |  |
| EU-12 | 6 | 2 | 5 | 1 | 70 | 93 |  |  |
| EU-13 |  | 2 | 2 | 1 |  |  |  |  |
| EU-14 |  | 2 |  |  |  |  |  |  |
| EU-16 |  |  |  | 1 |  |  |  |  |
| EU-17 | 2 | 1 | 2 |  |  |  |  |  |
| EU-18 | 1 | 2 | 1 |  |  |  |  |  |
| EU-19 |  |  | 1 |  |  |  |  |  |
| EU-21 |  | 2 |  |  |  |  |  |  |
| EU-22 | 1 | 3 | 1 | 2 |  |  |  |  |
| EU-23 | 3 | 1 |  |  |  |  |  |  |
| EU-25 |  | 1 |  | 1 |  |  |  |  |
| EU-26 |  | 3 | 1 |  |  |  |  |  |
| EU-27 | 1 |  | 1 |  |  |  |  |  |
| EU-28 | 1 | 1 |  |  |  |  |  |  |
| EU-29 | 1 | 3 |  |  |  |  |  |  |
| EU-31 |  | 1 |  | 1 |  |  |  |  |
| EU-43 |  | 1 |  |  |  |  |  |  |

**Table S2** Pairwise values for linearized F_ST_ (above diagonal) and geographic distance (below diagonal) used in the Mantel test for significance of isolation-by-distance (IBD) between populations of *Cryphonectria hypovirus 1* subtype I.

|  | Go | Pu | Ka | Iv | Ra | He | Bu | Ya |
| --- | --- | --- | --- | --- | --- | --- | --- | --- |
| Go | 0 | 0.00560 | 0.01843 | 0.01136 | 0.00463 | 0.03925 | 0.00426 | 0.01413 |
| Pu | 33 | 0 | 0.01715 | 0.01041 | 0.00368 | 0.03847 | 0.00329 | 0.01317 |
| Ka | 847 | 860 | 0 | 0.02359 | 0.01640 | 0.05237 | 0.01621 | 0.02683 |
| Iv | 906 | 918 | 65 | 0 | 0.00936 | 0.04517 | 0.00905 | 0.01935 |
| Ra | 1403 | 1408 | 623 | 559 | 0 | 0.03705 | 0.00230 | 0.01206 |
| He | 1732 | 1736 | 951 | 886 | 333 | 0 | 0.03738 | 0.04938 |
| Bu | 2311 | 2320 | 1475 | 1411 | 939 | 661 | 0 | 0.01178 |
| Ya | 2326 | 2336 | 1488 | 1425 | 959 | 686 | 31 | 0 |
